# A Recommender for Research Collaborators Using Graph Neural Networks

**DOI:** 10.3389/frai.2022.881704

**Published:** 2022-08-01

**Authors:** Jie Zhu, Ashraf Yaseen

**Affiliations:** Department of Biostatistics and Data Science, School of Public Health, The University of Texas Health Science Center, Houston, TX, United States

**Keywords:** graph neural networks (GNN), recommendation systems, collaborator recommendation, deep learning, artificial intelligence

## Abstract

As most great discoveries and advancements in science and technology invariably involve the cooperation of a group of researchers, effective collaboration is the key factor. Nevertheless, finding suitable scholars and researchers to work with is challenging and, mostly, time-consuming for many. A recommender who is capable of finding and recommending collaborators would prove helpful. In this work, we utilized a life science and biomedical research database, i.e., MEDLINE, to develop a collaboration recommendation system based on novel graph neural networks, i.e., GraphSAGE and Temporal Graph Network, which can capture intrinsic, complex, and changing dependencies among researchers, including temporal user–user interactions. The baseline methods based on LightGCN and gradient boosting trees were also developed in this work for comparison. Internal automatic evaluations and external evaluations through end-users' ratings were conducted, and the results revealed that our graph neural networks recommender exhibits consistently encouraging results.

## Introduction

Academic collaboration can be termed as a research effort done by researchers either from different disciplines or from the same discipline across different groups, nationally or internationally (Katsouyanni, 2008). Collaborations between researchers often have a synergistic effect as follows: The combined expertise of a group of researchers can often yield results far surpassing the sum of the individual researchers' capabilities (Pavlov and Ichise, 2007). As important as it is, however, it is often challenging to find suitable collaborations, partly due to the sheer number of researchers out there and the fact that it is impractical for anyone to be fully aware of others' expertise within a limited time, if they have not known each other beforehand, especially for junior scholars. Adding to that, the constant change is researchers' expertise, areas of interest, and knowledge over time (more rapid for some researchers than others), all make it more challenging. Recommendation systems (RS), or recommenders, have proven to be an effective strategy to deal with such information overload.

Recommendation systems are information filtering systems that deploy data mining and analytics of users' behaviors, for predictions of users' interests in information, products, or services. Given their wide and successful use in commercial applications, RS is being explored and applied in the academic domain as well (Zhu et al., 2021).

In this work, we focused on the following two Graph Neural Networks (GNN) models that cater to our collaborator recommendation problems: GraphSAGE (Hamilton et al., 2017) and Temporal Graph Networks (TGN; Rossi et al., 2020). Both GraphSAGE and TGN are inductive GNNs that are able to generate embeddings for unseen nodes, in addition, TGN was specifically designed for graphs that are dynamic in nature. With their state-of-the-art performances on several transductive and inductive graph prediction tasks, we built on these foundations for recommending collaborators in the population health domain to study this under-explored area. We carefully designed and executed a series of experiments to develop our collaborators recommender. The evaluations of the experiments on the recommender were conducted both internally (using existing relationships within the data) and externally (by collecting end-users' ratings). Our main contributions are as follows:

- We employed novel inductive GNN networks (GraphSAGE and TGN), instead of the traditional machine learning techniques and transductive-only GNNs, to capture intrinsic, complex, and temporal dependencies among researchers for future link predictions.
- Unlike other GNNs commonly used for predicting static user–item interactions in the industry domain (e.g., restaurants, music, and movies to people), we focused on the temporal user–user recommendations in the academic domain.
- We crawled the following data suitable for real-world applications: Datasets from the MEDLINE database with a wide range of time periods and achieved consistently encouraging results for predictions.
- Finally, we developed a web-based application for our recommender, available at http://genestudy.org/recommends/#/collaborators, giving our research practical use. This also allowed us to collect feedback/ratings from end-users to conduct an external evaluation of the system.

The rest of the content in this manuscript is organized as follows: the “Related work” section summarizes the available literature on the topic of collaboration recommendation; the “Materials and Methods” section presents the data and methods we used and the evaluations we carried out for two scenarios of experiments; “Results” section summarizes our GNNs performances; and finally, “Discussion and conclusion” section reiterates our main contributions, extends an in-depth analysis of the results, and brings forward our future plans for the continuation of this work.

## Related Work

Collaboration recommendation problems have been commonly addressed in the context of link predictions within networks. The link predictions aim to predict the likelihood of a future association (in our case, collaborations) between two nodes (in our case, researchers), based on the current states of links and nodes in the network (Yu et al., 2014). However, most of the link predictions have been solved using the network topological features for nodes in concern and the Markov process (random process index by time in the topological space), or a combination of both methods. Liben-Nowell and Kleinberg (2007) were the first that applied the topological features of node similarities measures toward linkage predictions, measures that they systematically compared included shortest path, common neighbors, preferential attachments, and six others. Afolabi et al. (2021) used the simple Adamic–Adar Index (Adamic and Adar, 2003) for the link predictions among computer science researchers. Pavlov and Ichise (2007) utilized multiple structural attributes, such as the shortest path from past collaborations, coupled with supervised machine learning algorithms, i.e., support vector machines (SVM) and decision trees, for predicting future research potentials. Yu et al. (2014) also utilized several structural attributes with logistic regression and SVM for collaboration recommendations in the medical domain. Lopes et al. (2010) employed topic modeling to build the profiles of researchers and the asymmetric variant of Jaccard' coefficients (Salton and McGill, 1983) for calculating the node proximity. Cho and Yu (2018) used node similarity scores with multi-networks as follows: Co-authorship, researcher–journal, and school networks for the collaboration predictions. Wang and Sukthankar (2013) introduced social features with “edge clustering” in addition to nodes' proximity measures (e.g., common neighbors, etc.) and employed machine learning models, i.e., Naïve Bayes, logistic regression, etc. for the collaboration predicting on DBLP dataset (Yang and Leskovec, 2012). Kuzmin et al. (2016) used the PageRank (Page et al., 1999), a random walk model, and multilayer networks that merged molecular interaction networks with authorship information for recommending biomedical collaboration potentials. Kong et al. (2016) adopted word2vec to identify the academic domains and the random walk with a restart to compute researchers' feature vectors for collaborator recommendations. Liu et al. (2018) combined neural networks based collaborative entity embedding with a hierarchical factorization model to produce context–aware collaborator results. However, there are still concerns about whether these techniques adequately and properly addressed the linkage issues. On the one hand, instead of relying on these hand-engineered topographical features or the Markov process, we are able to find an algorithm to capture the various information within the data elegantly and expressively for linkage predictions; on the other hand, more importantly, collaboration networks, in general, are not static; researchers and their associations evolve dynamically over time, and therefore ideal algorithms should capture change and iteratively be updated with new information, handle emerging researchers, and recommend potential collaborations accordingly.

With the advances in deep learning (DL), DL models have been popular methodologies for recommenders due to their ability to capture non-linear/complex relationships in data and easily incorporate temporal, contextual, and external information (Mu, 2018). Among these DL algorithms, GNN is undoubtedly the most attractive one due to its superior ability on graph/network structured data to combine connectivity and features of local neighbors, which fits naturally with recommenders' link predictions (Ying et al., 2018; Wu S. et al., 2020). The main idea of GNN is to iteratively aggregate feature information from local neighbors and integrate the aggregated information with the current node representation during the propagation process (Wu Z. et al., 2020; Zhou et al., 2020). There have been some efforts to use GNN for the link predictions (Zheng et al., 2018; Fan et al., 2019; Wang et al., 2019; Zhang et al., 2019; Chen et al., 2020; He et al., 2020; Sun et al., 2020; Zhang and Chen, 2020; Mandal and Maiti, 2021); nevertheless, they were focusing on encoding the static collaborative signal of bipartite user–item interactions instead of the evolving social user–user relationships. Among these, some of the following studies modified the graph operations to be more suitable for collaborative filtering: Chen et al. (2020) removed non-linearity and introduced residual network structures; LightGCN (He et al., 2020) was proposed for learning user–item interactions without feature transformation or non-linear activation; Zheng et al. (2018) introduced spectral convolution operation to discover and learn from the deep connections of user–item in the spectral domain; Zhang et al. (2019) developed a stacked GCN encoder–decoder architecture to solve the user–item matrix completion problem; Zhang and Chen (2020) extracted h-hop enclosing subgraphs to train a GNN for rating matrix completion. Some utilized fixed hierarchical bipartite graphs on the user–item interactions to produce embeddings: Wang et al. (2019) proposed Neural Graph Collaborative Filtering, a framework that explicitly encodes the user–item signal in the form of high-order connectivity using embedding propagation; Mandal and Maiti (2021) incorporated the degree of authenticity of reviews and customers in their trust-based social recommendation, a system which is composed of both customers networks and customer–item interaction networks. Sun et al. (2020) introduced a pairwise neighborhood aggregation layer to explicitly model the relational information between the neighbor nodes, and developed parallel GCNs to exploit the heterogeneous nature of the user–item bipartite graph for recommendation; Fan et al. (2019) developed GraphRec, which jointly captured the interactions and opinions in the user–item graph and heterogeneous social graph.

## Materials and Methods

### Experiment Scenario With Automatic Evaluations

**Figure 1:**
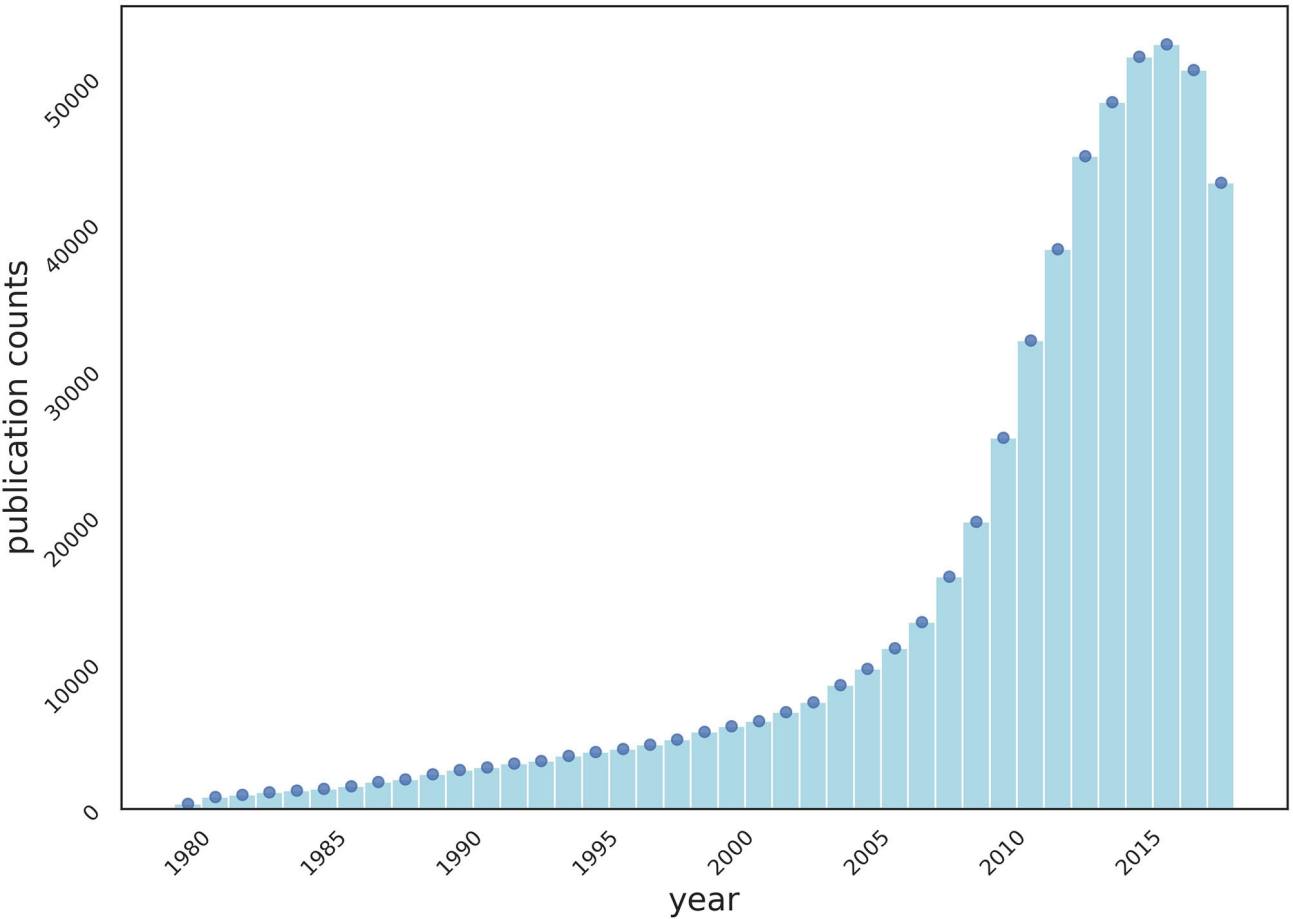
Publication counts through the years 1980–2020 in crawled MEDLINE dataset.

**Figure 2:**
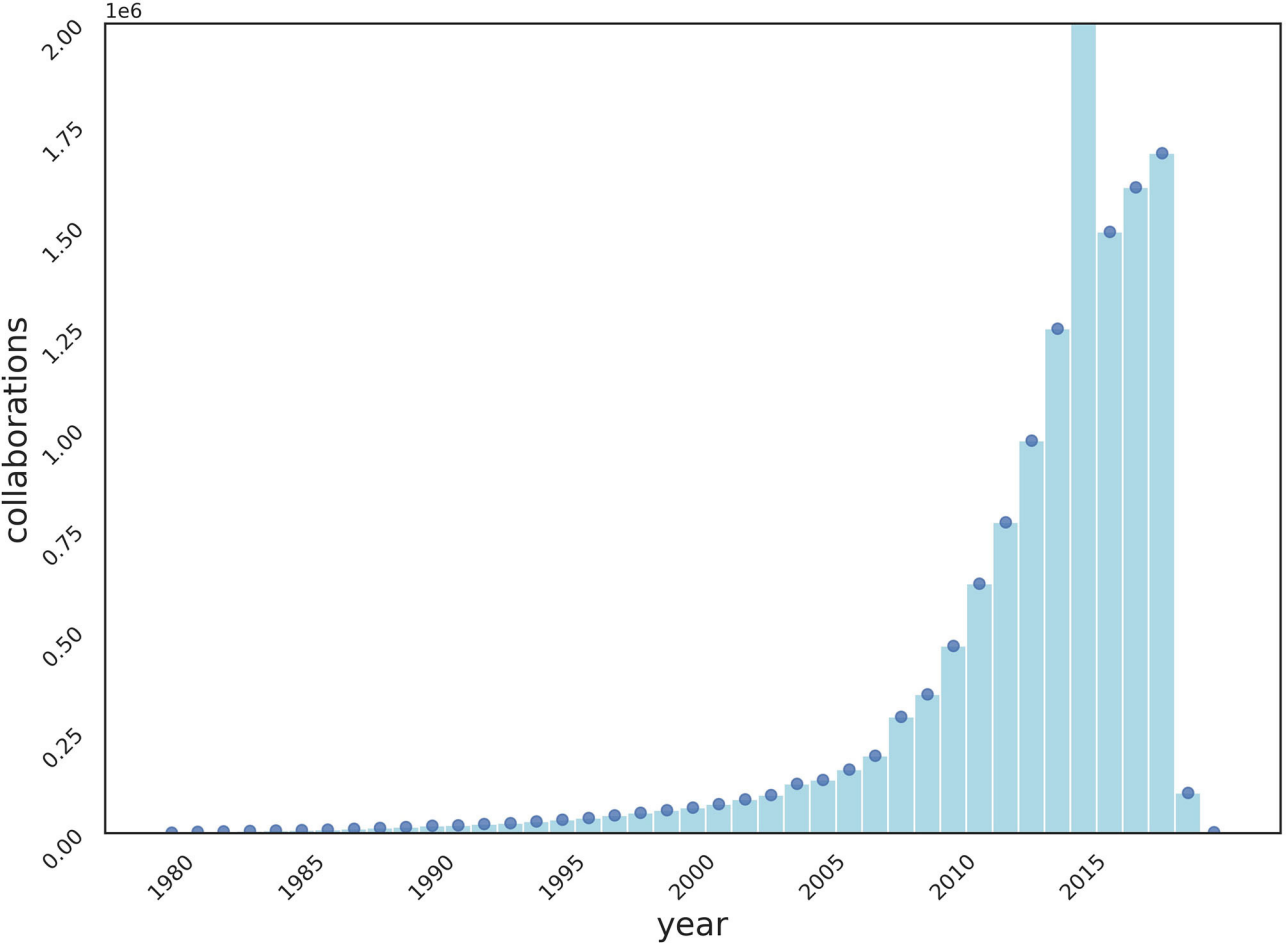
Collaboration counts through the years 1980–2020 in crawled MEDLINE dataset.

We crawled MEDLINE, a public dataset of 33 million citations, from PubMed^1^, for the experiments of collaboration recommendations. Collaborations are defined as “two or more authors sharing a publication.” The number of publications (Figure 1) and collaborations (Figure 2) are surging each year.

We followed similar practices in the existing literature on linkage predictions (Pavlov and Ichise, 2007; Yu et al., 2014; Chuan et al., 2018) and sampled three time periods from the dataset to cover a wider time range. The three time periods are 2000–2002, 2010–2011, and 2019–2020. All follow the 70:15:15% train, validation, and test chronological split as discussed in Rossi et al. (2020) and Xu et al. (2020). For validation and test, inductive tasks (Hamilton et al., 2017) refer to the link predictions involving nodes that have never appeared during graph training. The GNN models were trained and ultimately evaluated on test data and an inductive subset^2^ of test data, while baseline methods were evaluated on the whole test data (because of its inability to perform in inductive tasks). The detailed statistics of the three sets of data are described in Table 1.

**Table 1:** Dataset statistics for three experimental settings.

**Time period**	**Splits**	**Number of** **links**	**Number** **of nodes**
2000–2002	Training	127,040	32,656
	Validation, All	32,053	10,150
	*Validation, inductive subset*	*26,989*	*9,757*
	Test, All	32,342	8, 825
	*Test, inductive subset*	*28,417*	*8,548*
	**Total**	**215,815**	**44,607**
2010–2011	Training	418,641	77,048
	Validation, All	103,386	24,984
	*Validation, inductive subset*	*88,077*	*24,386*
	Test, All	104,116	23,055
	*Test, inductive subset*	*89,633*	*22,529*
	**Total**	**694,568**	**108,594**
2019-2020	Training	67,246	11,960
	Validation, All	16,653	1804
	*Validation, inductive subset*	*16,378*	*1803*
	Test, All	13,178	2,519
	*Test, inductive subset*	*12,968*	*2,519*
	**Total**	**101,126**	**15,853**

*(Dataset statistics for three experimental settings), totals for each time period are shown in bold*.

We experimented with two GNN methods (GraphSAGE and TGN) for producing node embeddings and attached a small neural network to feed on produced embedding for linkage predictions. We compared the performances with a transductive GNN method; LightGCN and a machine learning method; Gradient boosted classifier.

#### GraphSAGE

GraphSAGE (Hamilton et al., 2017) is a framework for inductive representation learning on large graphs. Specifically, GraphSAGE generates embeddings for each node (including unseen ones) by (1) uniformly sampling a fixed small number of neighbors and aggregating the neighbor embeddings using learnable aggregators, such as mean, graph convolution networks (GCN); (2) concatenating neighbor embeddings with the embedding of the node itself; (3) feeding the concatenated embeddings to fully connected networks, represented by the following equation:


$$\begin{array}{l} h_{v}^{k}=\;\sigma \left(\;W_{k}•concat\left(agg\left(\left\{h_{u}^{k-1},\;\forall \;u\in N\left(v\right)\right\}\right),\;h_{v}^{k-1}\right)\right) \end{array}$$

where $h_{v}^{k}$ is the embeddings for node *v* at *k*th iteration; *N*(*v*): neighbors of node *v*; $h_{u}^{k-1}\;$: the neighbor embeddings at *k*th iteration; σ: activation function; and ***W***_*k*_: weight matrix.

**Figure 3:**
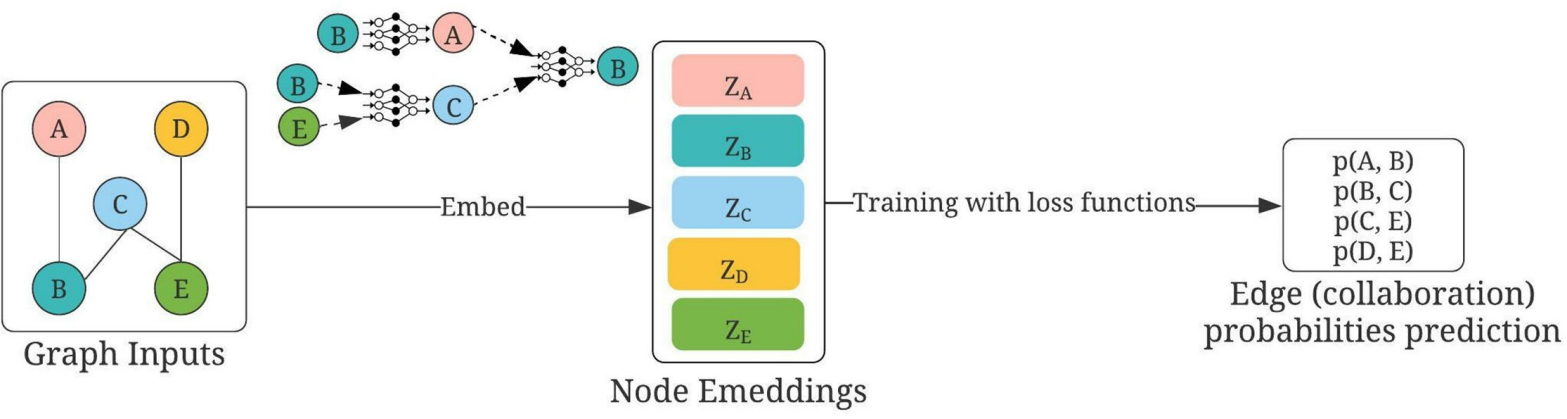
Usage of GraphSAGE for collaborator recommendations, modified based on Hamilton et al. (2017). Nodes A, B, C, D, and E are authors; first constructed by using either (1) mesh terms or (2) titles of publications. Links are defined by “sharing of publications.” During training, the embeddings of each node are updated using GraphSAGE, as detailed in the text. Then the question of collaboration predictions becomes as follows: Given the embeddings of two nodes, how likely will there be a link between them?

**Figure 4:**
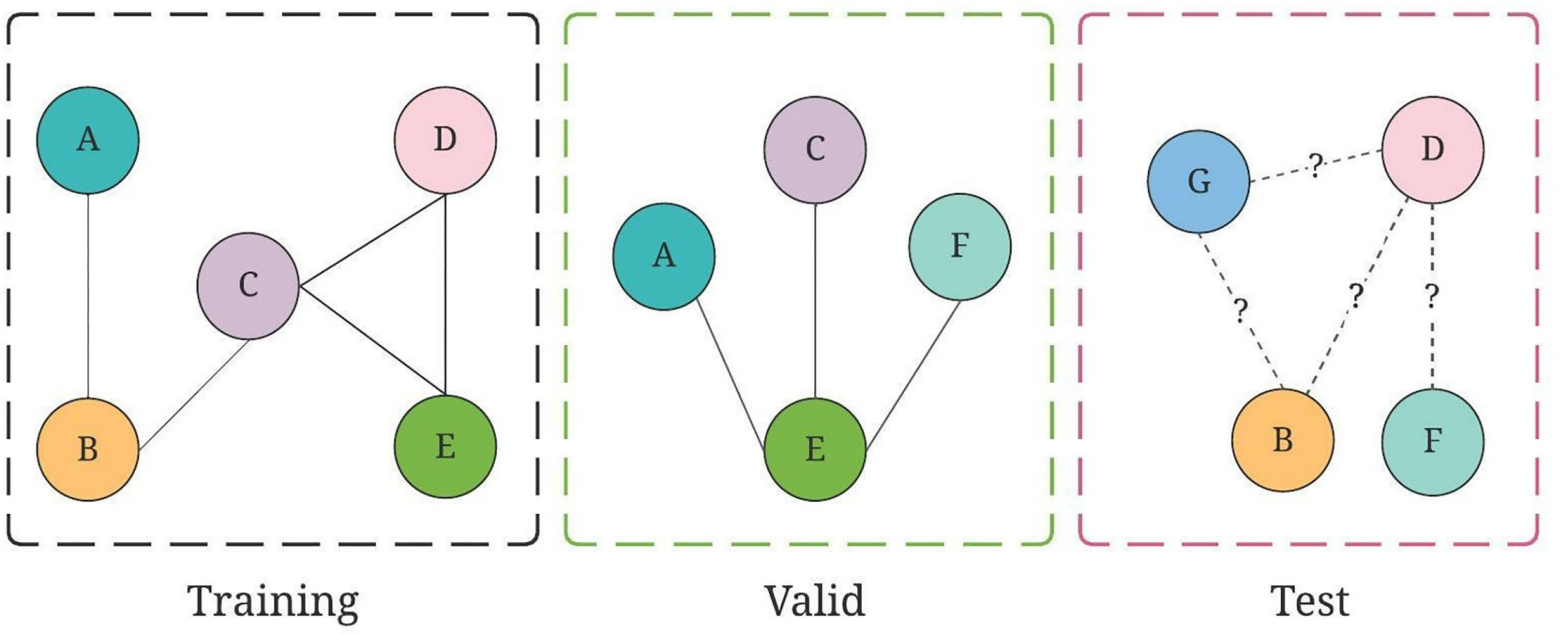
An illustration of training, valid, and test graph construction for GraphSAGE, where collaborations are split on chronological order.

We defined the nodes as authors and links as collaborations, with the collaborations defined as “the two authors sharing an article” as stated earlier. We constructed the raw node features using Term Frequency–Inverse Document Frequency (TF–IDF) with the following two variations: (1) Mesh terms of the articles (Mesh or Medical Subject Headings^3^, is the National Library of Medicine controlled vocabulary thesaurus used for indexing articles for PubMed and (2) titles of the articles. The usage is illustrated in Figure 3. The training, validation, and test graph construction for GraphSAGE can be found in Figure 4. We used the Pytorch implementation of GraphSAGE by Deep Graph Library^4^. The detailed hyperparameters used can be found in Table 2.

**Table 2:** Hyperparameters used for training GraphSAGE.

		**Value**
Model architecture	Number of layers	2
	Node embedding dimensions	200
	Pooling methods	GCN, GCN
	Dropout	0.
Training parameters	Learning rate	0.005
	Optimizer	Adam
	Epochs	100
	Number of GPU	1

#### Temporal Graph Networks

Temporal graph networks (Rossi et al., 2020) is a generic framework for deep learning on dynamic graphs represented as sequences of timed events, which produces the embeddings of graph nodes ***Z***(*t*) = (***z***_1_(*t*), …, ***z***_*n*(*t*)_(*t*)). There are four important components that constitute the TGN, which are message function, memory, memory updater, and embeddings. Message functions compute two messages for the involving nodes (*i, j*) using the memories of *i, j* before the link, the link timestamp, and edge features, if any. Memory modules store the states of all nodes up till time *t*:*s*_*i*_(*t*), acting as the compressed representations of the nodes' past interactions. Memory updater updates the memory with the new messages. Finally, the embedding module computes the temporal embeddings of the nodes by performing a graph aggregation over the spatial–temporal neighbors of the node in concern, similar to GraphSAGE.

**Message function:**

$m_{i}\left(t\right)\;[or\;m_{j}\left(t\right)]=msg[s_{i}\left(t^{-}\right),\;s_{j}\left(t^{-}\right),\;△t,\;e_{i,j}\left(t\right)],$

${\bar{m}}_{i}\left(t\right)=agg\left[m_{i}\left(t_{1}\right),\ldots \;m_{i}\left(t_{b}\right)\right]$,
where *t*_1_, …, *t*_*b*_ ≤ *t*.
***m***_*i*_(*t*) is the message for node *i* at time *t*; $s_{i}\left(t^{-}\right)$ is the memory of node *i* before time *t*; *e*_*i, j*_(*t*) is the edge features of node *i, j* at time *t*; ${\bar{m}}_{i}\left(t\right)\;$is the aggregated message for node *i* at time *t* after applying an aggregator function on the past messages for node *i*.
**Memory updater:** $s_{i}\left(t\right)=memfunc\left[{\bar{m}}_{i}\left(t\right),\;s_{i}\left(t^{-}\;\right)\right]$
where *memfunc* is a learnable memory update function, such as GRU.
**Embedding:** $z_{i}\left(t\right)=\;\underset{j\in N_{i}\left[0,\;t\right]}{\sum }h\left[s_{i}\left(t\right),\;s_{j}\left(t\right),\;e_{ij}\left(t\;\right)\right]$
where *h* is a learnable function such as graph attention (Rossi et al., 2020), *N*_*i*_[0, *t*] is node *i*'s neighbors up to time *t*.

**Figure 5:**
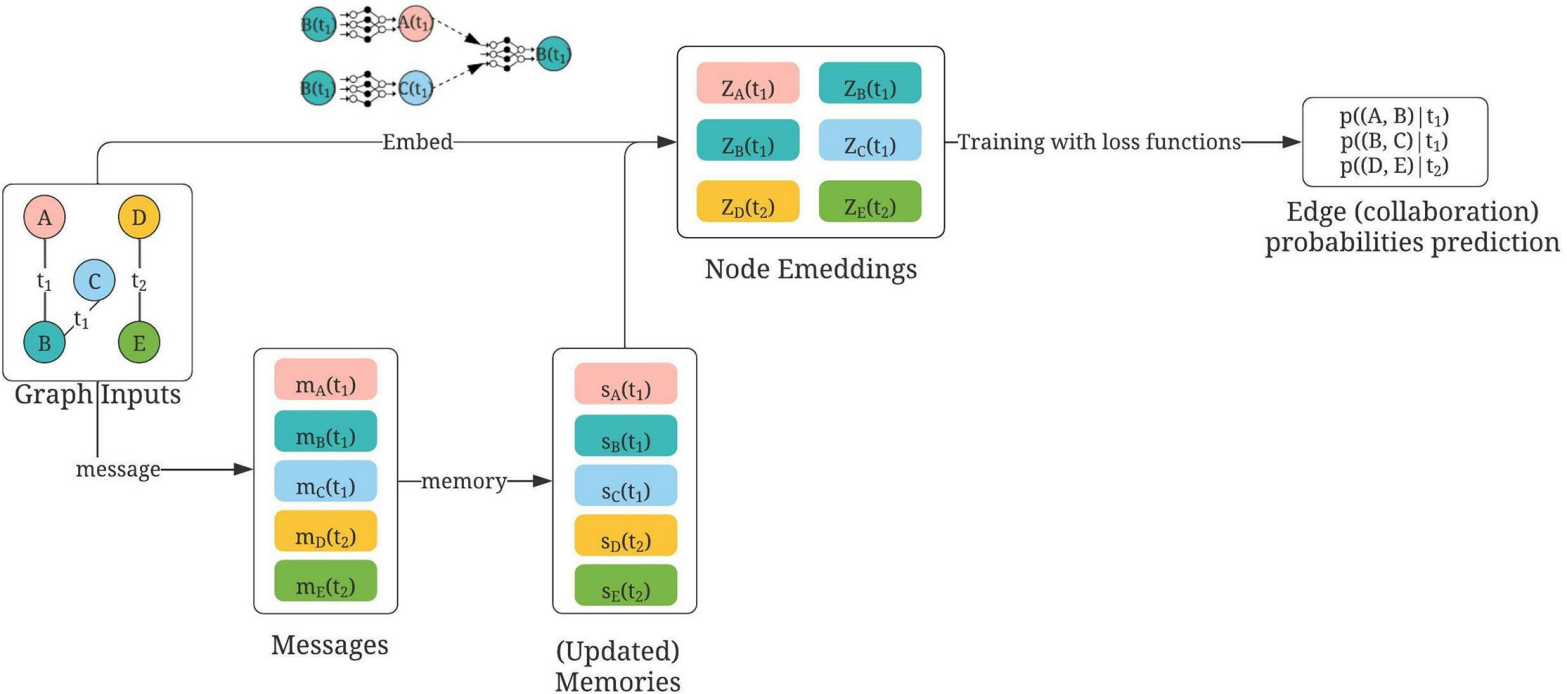
Usage of TGN for collaborator recommendations, modified based on Rossi et al. (2020). Nodes A, B, C, D, and E are authors; first constructed by using either (1) mesh terms or (2) titles of publications. Temporal links are defined by “sharing of publications” at time *t*. During training, each temporal collaboration is computed within a message between the involved nodes [e.g., at time *t*_2_, the collaboration between D and E is calculated in both messages, ***m***_*D*_(*t*_2_) and ***m***_*E*_(*t*_2_)]. Then the memory state of each author is updated using those temporal messages. The updated memories, together with embeddings constructed similar to GraphSAGE (concatenation of neighbor embeddings and its own embeddings) are then aggregated as the final embedding for each node. Then the question of collaboration predictions becomes as follows: Given the embeddings of two nodes at time *t*, how likely will there be a link between them?

**Figure 6:**
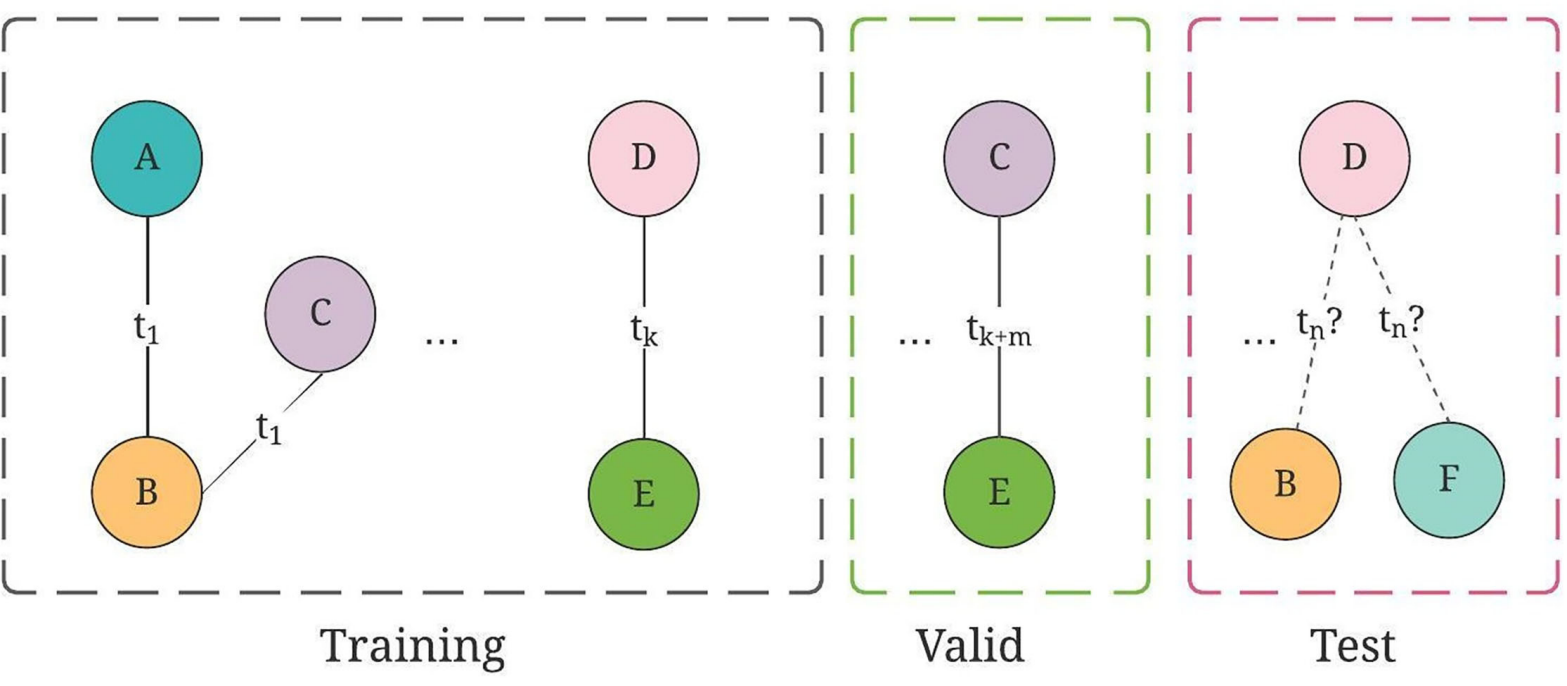
An illustration of training, valid, and test graph construction for TGN, where the collaborations are split on chronological order.

The graph was defined similarly to the way used in GraphSAGE, with the timestamp of the links explicitly represented using the publication date. The usage is illustrated in Figure 5. The training, validation, and test graph construction for TGN can be found in Figure 6. We modified the architecture based on the original GitHub repo by twitter research^5^. The detailed hyperparameters used can be found in Table 3.

**Table 3:** Hyperparameters used for training TGN.

		**Value**
Model architecture	Number of layers	1
	Node embedding dimension	200
	Number of attention heads	2
	Message dimension	50
	Memory dimension	172
	Memory updater	GRU
	Dropout	0.1
Training parameters	Learning rate	0.0001
	Optimizer	Adam
	Epochs	100
	Number of GPU	1

#### Baseline: LightGCN

As a competitive transductive GNN baseline, LightGCN was chosen because of its efficiency in many static and transductive recommendation tasks (He et al., 2020; Ragesh et al., 2021). The most essential part of this model is a simplified graph convolution with neither feature transformations nor non-linear activations. The weighted sum of embeddings learned at different propagations layers was used for final node embeddings. Similar to GraphSAGE and TGN-based recommenders, the original features of each author were constructed using mesh terms first, then the titles of the articles. We used the implementation of LightGCN from Pytorch Geometric^6^ with hyperparameters as detailed in Table 4.

**Table 4:** Hyperparameters used for training LightGCN baseline.

		**Value**
Model architecture	Number of layers	2
	Node embedding dimension	200
Training parameters	Learning rate	0.005
	Optimizer	Adam
	Epochs	100
	Number of GPU	1

#### Baseline: Gradient Boosted Classifier (GBC)

Finally, GBC was chosen as another baseline because of its simplicity in implementation, scalability, and proven successes in many competitions (Chen and Guestrin, 2016), in consideration of bias–variance trade-off (Hastie et al., 2009). Simply, boosting is a type of ensemble method that builds weak/slow learners (usually trees) sequentially, to correct the previously wrongly predicted outcomes. Similar to GraphSAGE and TGN, the feature space of each author was constructed using mesh terms and titles of the articles separately for two scenarios. We used the implementation from XGboost^7^, hyperparameters are detailed in Table 5.

**Table 5:** Hyperparameters used for training GBC baseline.

		**Value**
Model architecture	Number of trees	100
Training parameters	Learning rate	0.05
	Epochs	100
	Number of GPU	1

#### Evaluation Metrics

For all four methods, we used ROC–AUC (AUC) and average precision (AP) for evaluations, which were widely used in experiments (Yu et al., 2014; Rossi et al., 2020; Xu et al., 2020). Since GraphSAGE and TGN are able to handle new nodes, the two metrics were also used to evaluate the inductive subset of the test data.

- AUC: A receiver operating characteristic (ROC) curve is a graphical plot that illustrates the diagnostic ability of a binary classifier system as its discrimination threshold is varied; AUC is the area under an ROC curve, which provides an aggregated measure of performance across all possible classification thresholds (Fawcett, 2006).
- AP: It summarizes precision–recall curve as the weighted mean of precisions achieved at each threshold, with the increase in recall from the previous threshold used as the weight.

### Experiment Scenario With External Evaluations

In addition to using existing relationships in crawled data, we further explored the external evaluations by collecting ratings and feedback directly from end-users. We used their PubMed publications available from their resumes from 2019 to 2021 and predicted the possibilities of future collaborations between the user and all authors in the existing database that the user had not interacted with yet.

**Figure 7:**
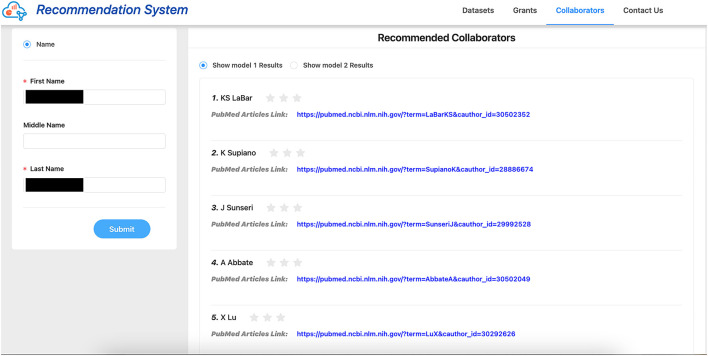
Screenshot of external evaluation page.

We provided 1–3 stars for all users to rate our collaborator recommendations, with 3 stars being “the most satisfactory” and 1 star being “the least satisfactory.” See Figure 7 for an example of the evaluation collection page. We considered the recommendations with 2 stars and above as “relevant recommendations,” thus precision@k is calculated as follows: at the *k*th retrieved item, the proportion of the retrieved items are relevant. We look at both *k* = 1 and *k* = 5.


$$\begin{array}{l} precision@k\;=\frac{TP@k}{TP@k+FP@k} \end{array}$$

The codes for all current experiments can be found at the GitHub repo: https://github.com/ashraf-yaseen/VRA/tree/master/collaborator_rec.

## Results

For the experiments with automatic evaluations, the AUC and AP for two GNN-based methods *vs*. baselines are shown in Tables 6, 7; all results were averaged over 5 runs. Node/author features for two tables were constructed using mesh terms (Table 6) and publication titles (Table 7) respectively, as detailed in the “Related work” section.

**Table 6:** AUC and AP for GraphSAGE, TGN, and baseline LightGCN, baseline GBC on sampled datasets from three time periods using mesh terms as node features, with best performances shown in bold.

**Time frame**	**Models**	**Test AUC-all**	**Test AUC-inductive** **subset**	**Test AP-all**	**Test AP-inductive subset**
2000–2002	GraphSAGE	0.774	**0.770**	0.747	**0.739**
	TGN	0.803	0.675	0.830	0.706
	LightGCN	**0.862**	NA	**0.868**	NA
	GBC	0.752	NA	0.678	NA
2010–2011	GraphSAGE	0.752	0.757	0.712	**0.718**
	TGN	0.780	**0.824**	0.599	0.682
	LightGCN	**0.921**	NA	**0.933**	NA
	GBC	0.764	NA	0.745	NA
2019–2020	GraphSAGE	0.728	**0.735**	0.667	**0.672**
	TGN	0.854	0.621	0.864	0.656
	LightGCN	**0.920**	NA	**0.928**	NA
	GBC	0.640	NA	0.619	NA

**Table 7:** AUC and AP for GraphSAGE, TGN, and baseline LightGCN, baseline GBC on sampled datasets from three time periods using article titles as node features, with best performances shown in bold.

**Time frame**	**Models**	**Test AUC-all**	**Test AUC-** **inductive** **subset**	**Test AP-all**	**Test AP- inductive** **subset**
2000–2002	GraphSAGE	0.771	0.776	0.747	0.757
	TGN	**0.962**	**0.934**	**0.965**	**0.941**
	LightGCN	0.864	NA	0.869	NA
	GBC	0.792	NA	0.729	NA
2010–2011	GraphSAGE	0.770	0.785	0.734	0.761
	TGN	**0.972**	**0.947**	**0.975**	**0.956**
	LightGCN	0.918	NA	0.924	NA
	GBC	0.801	NA	0.767	NA
2019–2020	GraphSAGE	0.766	0.773	0.734	0.741
	TGN	**0.928**	**0.883**	**0.944**	**0.906**
	LightGCN	0.917	NA	0.926	NA
	GBC	0.724	NA	0.713	NA

When constructing node/author features using mesh terms, we can see that the best transductive performances were all achieved by LightGCN, demonstrating its competitive ability in learning linkage representations when node features were confined. For inductive subsets, the best performances were split between GraphSAGE and TGN.

When constructing node/author features using publication titles, however, TGN was able to achieve consistently the best results, both transductive and inductive, across all time periods. Furthermore, we can see varying performance improvements over all three methods except for the LightGCN baseline as discussed in the following: A significant improvement on TGN (maximum 62.8% improvement on test AP), a moderate improvement on GraphSAGE (maximum 10.3% improvement on test AP, inductive subset) and on GBC baseline (maximum 15.2% improvement on test AP), and finally a small fluctuation on LightGCN (maximum 0.23% improvement on test AUC, and maximum 0.96% deterioration on test AP). Since the publication titles capture richer information than the mesh terms, this showed that when the raw node features were provided well, TGN was better fitted for the linkage predictions in addition to its inductive ability.

Given this improved performance when using publication titles as node features, we decided to adopt this representation to implement our GraphSAGE and TGN-based recommenders. The two methods were then deployed within a web-based application to allow for external evaluations. We asked several researchers in our institute to evaluate the recommendations of the two methods. At this time, we received ratings/feedback from seven users. Simple statistics of their publications, the average stars, P@1, and P@5 for both GraphSAGE and TGN are shown in Table 8.

**Table 8:** External evaluations collected from users, with “**” indicating the existence of 3-star rating.

**Users**	**Number of articles** **for experimentation**	**GraphSAGE**			**TGN**		
		**Average** **stars**	**P@1**	**P@5**	**Average** **stars**	**P@1**	**P@5**
User 1	7	2.0	1.(**)	0.8	2.1	1.(**)	1.
User 2	4	1.8	1.	0.6	2.0	1.	1.(**)
User 3	10	1.2	0.	0.2	1.0	0.	0.
User 4	7	1.2	0.	0.2	1.2	0.	0.2
User 5	15	1.1	1.	0.2	1.1	0.	0.2
User 6	12	1.0	0.	0.	1.6	0.	0.2(**)
User 7	21	1.0	0.	0.	1.0	0.	0.
Average	11	1.3	0.43	0.29	1.4	0.29	0.37

Overall, we can see that GraphSAGE produced better top 1 hits than TGN (P@1 = 0.43 vs. 0.29), but less top-5 hits in general (P@5 = 0.29 vs. 0.37). However, TGN was able to make more strictly relevant recommendations (3-star rating) than GraphSAGE. The external evaluations are inherently more subjective and thus are not idealistic as the automatic evaluations. Considering this, we think that TGN especially has the potential to deliver satisfactory recommendations with proper adjustments, and it will be the foundational architecture of our collaborator recommender moving forward. Nevertheless, we do acknowledge that this is only a small sample evaluation due to low response rates (30%), and therefore larger sample evaluation should be carefully carried out for analysis in the future.

## Discussion and Conclusion

In this work, a collaboration recommendation system using novel GNNs (GraphSAGE and TGN) have been developed. Moreover, baselines using both transductive GNN and GBC have also been developed for comparison. The ability of our method to capture intrinsic, complex, and changing dependencies among researchers, including temporal user–user interactions is crucial and important to the overall performance of the recommender. The internal evaluations using crawled collaborators networks revealed that our TGN, when well-supplied with node features, consistently exhibited better performance compared with the baselines, in addition to its strong inductive ability. The external evaluations also revealed that our models are of practical value with encouraging P@1 and P@5 on a small sample, which, we hope, could prove useful to the population health professionals on a larger scale with proper adjustments.

Furthermore, we believe that there is still room for improvement. First, in terms of raw node features, it would be interesting to investigate the possibility of presenting each author as a distribution of features instead of a fixed vector. Representing data with a distribution comes with many advantages; for example, it can allow better uncertainty encodings, and express asymmetries more naturally than dot product, cosine similarity, or Euclidean distance (Wu S. et al., 2020). Managing the uncertainty that is inherent in RS for predictive modeling is important for us to understand how we should interpret our recommendation results and act accordingly. In fact, many popular techniques in RS such as Upper Confidence Band (Auer et al., 2002) and Thomas sampling (Thompson, 1933) require uncertainty estimation to perform more efficient feature space exploration (Zeldes et al., 2017). Dos Santos et al. (2017) and Jiang et al. (2019) actually deployed Gaussian embeddings to capture users' uncertain preferences for improving user representations and recommendation performance. However, in general, the distribution-based representations have not been well-studied in the GNN-based recommendation models (Wu S. et al., 2020). Thus, we hope to work on modifying existing graph architectures in the future to add to this knowledge.

Second, in this work, GNN models were tuned with a random search on the validation dataset for the important hyperparameters centered on reported “best performing” values in the original articles (number of layers, embedding dimensions, learning rate, etc.), due to the large number of hyperparameters involved, as well as corresponding lengthy time consumptions. However, more efficient and thorough hyperparameter tuning should be implemented and better examined for the completeness of the study.

Finally, a detailed look into the external evaluations found that both GNN models favored the researchers from publications with long author lists. This was not surprising, since a long author list can create more links for the graph constructions. One possible next step is to analyze how placing penalty weights on the publications with long authors' lists might affect the performance of our recommender. Another improvement would be to collect users' text feedback in addition to ratings, though this might result in an even lower response rate. Users' text reviews could reveal additional features to consider for encoding, such as geographical locations or affiliations of researchers that could make our recommendations more personalized, thereby more fitting in the service scenario.

## Data Availability Statement

The original contributions presented in the study are included in the article/supplementary material, further inquiries can be directed to the corresponding author.

## Author Contributions

JZ was responsible for the background review, methodology development, experiment conduction, results summary, discussion, and wrote the article with guidance from AY. AY supervised the concept discussion, contributed to the manuscript writing, and approved the submitted version. Both authors contributed to the article and approved the submitted version.

## Conflict of Interest

The authors declare that the research was conducted in the absence of any commercial or financial relationships that could be construed as a potential conflict of interest.

## Publisher's Note

All claims expressed in this article are solely those of the authors and do not necessarily represent those of their affiliated organizations, or those of the publisher, the editors and the reviewers. Any product that may be evaluated in this article, or claim that may be made by its manufacturer, is not guaranteed or endorsed by the publisher.

## Footnotes

^1^ https://pubmed.ncbi.nlm.nih.gov/

^2^Inductive subset: as long as one node (of the connecting two) of the link has never appeared in the training set. Also appeared as “inductive task” in the text earlier.

^3^ https://www.nlm.nih.gov/mesh/meshhome.html

^4^ https://www.dgl.ai/

^5^ https://github.com/twitter-research/tgn

^6^ https://pytorch-geometric.readthedocs.io/en/latest/

^7^ https://xgboost.readthedocs.io/en/latest/
